# Factors associated with latent tuberculosis among asylum seekers in Switzerland: a cross-sectional study in Vaud County

**DOI:** 10.1186/1471-2334-12-285

**Published:** 2012-11-02

**Authors:** Apostolos Sarivalasis, Jean-Pierre Zellweger, Mohamed Faouzi, Oscar Daher, Charlotte Deslarzes, Patrick Bodenmann

**Affiliations:** 1 Department of Ambulatory Care and Community Medicine (PMU), Rue du Bugnon 44, 1011, Lausanne, Vaud, Switzerland; 2 Swiss Lung Association, Vaud section (LPVD), Av de Provence 4, 1007, Lausanne, Vaud, Switzerland; 3 Institute of Social and Preventive Medicine (IUMSP), Rue du Bugnon 44, 1011, Lausanne, Vaud, Switzerland; 4 Health Center – Sainte-Croix Hospital (CSSC), Rue des Rosiers 29,1450 Ste-Croix, Vaud, Switzerland; 5 Nurse, Health Center (CSI), Rue du Bugnon 44, 1011, Lausanne, Vaud, Switzerland

**Keywords:** Asylum seeker, Latent tuberculosis infection, Tuberculosis, Risk factors, Predictive score, Interferon gamma release assay

## Abstract

**Background:**

Screening and treatment of latent tuberculosis infection (LTBI) in asylum seekers (AS) may prevent future cases of tuberculosis. As the screening with Interferon Gamma Release Assay (IGRA) is costly, the objective of this study was to assess which factors were associated with LTBI and to define a score allowing the selection of AS with the highest risk of LTBI.

**Methods:**

In across-sectional study, AS seekers recently arrived in Vaud County, after screening for tuberculosis at the border were offered screening for LTBI with T-SPOT.TB and questionnaire on potentially risk factors. The factors associated with LTBI were analyzed by univariate and multivariate regression.

**Results:**

Among 393 adult AS, 98 (24.93%) had a positive IGRA response, five of them with active tuberculosis previously undetected. Six factors associated with LTBI were identified in multivariate analysis: origin, travel conditions, marital status, cough, age and prior TB exposure. Their combination leads to a robust LTBI predictive score.

**Conclusions:**

The prevalence of LTBI and active tuberculosis in AS is high. A predictive score integrating six factors could identify the asylum seekers with the highest risk for LTBI.

## Background

Most of the asylum seekers entering in Switzerland lived in countries with higher incidence rate of tuberculosis than in Western Europe and have a high risk of latent tuberculosis infection (LTBI) [1]. Persons with LTBI are at risk of developing an active tuberculosis mostly during the first years after infection [2]. A preventive treatment of infected but asymptomatic individuals lowers the risk of reactivation of latent infection and therefore decreases the pool of future active tuberculosis in a population [3]. Those principles are the basis of screening protocols for the management of individuals exposed to patients with active tuberculosis. Since the majority of asylum seekers are young adults the presence of LTBI among them would point to a recent contamination. Screening migrants for LTBI and treating those at risk of reactivation has been proven to be effective [4,5]. As most cases of active tuberculosis among asylum seekers occur within 5 years of entering Western countries and are due to the reactivation of a LTBI, screening and preventive treatment of LTBI, may be a complement to the screening for active tuberculosis in destination countries [6].

The current screening procedure for asylum seekers at the Swiss border consists on a standardized questionnaire on symptoms associated with tuberculosis, history of contact or prior treatment for active TB and an evaluation of the risk associated with the incidence in the country of origin. Migrants with symptoms or high risk of active tuberculosis are assessed by a physician. Those who are asymptomatic are transferred in a local center in a county. Screening for LTBI with tuberculin skin test (TST) was used during several years but was suspended after a study demonstrated the limited implications of a positive test result, particularly the weak observance of the treatment for LTBI by physicians and asylum seekers with a positive TST [7].

The introduction of Interferon-Gamma Release Assays (IGRAs) as a screening tool for tuberculosis infection changed the concept of screening individuals exposed to active TB and potentially infected [8]. IGRAs are highly specific, not influenced by prior BCG vaccination or contact with most non-tuberculous mycobacteria. They therefore help the physician to restrict the need for further examinations and prescription of preventive treatment.

Screening asylum seekers born in countries with high prevalence of tuberculosis upon entering Switzerland with IGRA is limited by the cost of the test and by the low number of migrants eligible for preventive treatment. Since individuals with LTBI are asymptomatic a screening based on clinical approach is not an option. A better definition of the factors associated with LTBI and reactivation of infection among asylum seeker might lower the cost of the screening procedure. The screening with IGRA of the asylum seekers presenting those factors could be cost-effective since the proportion of positive IGRA in this group would be higher than of the rest of the asylum seeker population. A recent prospective study from The Netherlands confirms the high rate of positive IGRA test results in immigrants and demonstrates that immigrants with a positive test result have a much higher risk of developing tuberculosis within two years after entry, irrespective of age and origin [9]. The authors consider the possible usefulness of a preventive treatment in this population group.

Based on such an assumption, some countries have introduced a selective screening for groups of asylum seekers considered at high risk of LTBI and reactivation [10,11]. The aims of this pilot study were to assess the prevalence of LTBI among asylum seekers entering Vaud County and to define the factors associated with latent infection among them.

## Methods

Vaud County is host to 9% of the total Swiss asylum seeker population. A cross-sectional study was conducted in two host centers (Sainte-Croix and Crissier) where the asylum seekers have to stay after the initial screening for active TB at the border. For financial and practical reasons (turnover of entries of AS in Switzerland and staff holidays), sampling lasted 10 months from September 2009 to July 2010.

All participating individuals were volunteers aged above 16 years, lodging in Sainte – Croix and Crissier host centers. All were recently arrived in Switzerland and had already been screened at the border for active tuberculosis two to three months before. They received detailed information on the study goals and on tuberculosis infection and signed a written consent form, translated in English, French, German, Russian and Arabic. For those asylum seekers who did not understand those languages a live translation was provided. The study was approved by the ethic commission of the University of Lausanne.

Certified nurses on each of the host center interviewed the individuals about their origin, demographic, travel conditions and medical history. Individuals mentioning a previous tuberculosis treatment were excluded from the study and addressed to the local health center to be assessed by a physician. In asylum seekers without a previous history of tuberculosis, 10ml venous blood was taken for T-SPOT-TB. The blood tubes were addressed with same day post mail to the laboratory.

The asylum seeker population was divided in two groups, positive and negative, according to the T-SPOT.TB results. Cases with 6 to 8 spots were considered according to the Swiss recommendations, as positive. No case was indeterminate.

Asylum seekers with a positive T-SPOT.TB result were addressed to the local health center for medical assessment. A physician examined them, asking for medical history of active tuberculosis or TB contact, performed a clinical examination and ordered a chest X-ray. HIV screening was proposed to all positive individuals, and women were offered pregnancy tests.

The asylum seekers presenting with cough, compatible with tuberculosis symptoms or abnormal chest X-ray had a sputum examination and culture. All asylum seekers with active tuberculosis were treated and excluded from the study. All asylum seekers with positive T-SPOT.TB without signs suspect of active TB or abnormal X-ray were considered as carriers of LTBI and were offered a preventive treatment. The follow-up and feasibility of LTBI preventive treatment is presented in a different paper.

The statistical data analysis was performed using STATA 11.2 (College Station, Texas 77845 USA). The data were summarized as mean (sd) for the age and as number (percentage) for categorical data. Univariate logistic regression analysis was performed to assess the association of the demographic, immigration history and medical factors associated with LTBI. Significant predictors at the level of 20% were used in a backward procedure to elaborate a multivariate model and to develop a prognostic scoring system to predict LTBI cases.

The β coefficients (β =log (OR)) derived from the multivariate logistic regression model were used to develop an overall prognostic scoring system. To simplify the calculation of the score, each β- coefficients were multiplied by 3 and were rounded to the closest integer. The score was calculated as the sum of the weighted scores from the six related factors. The discriminatory power of the score was assessed with a ROC curve.

## Results

**Figure 1:**
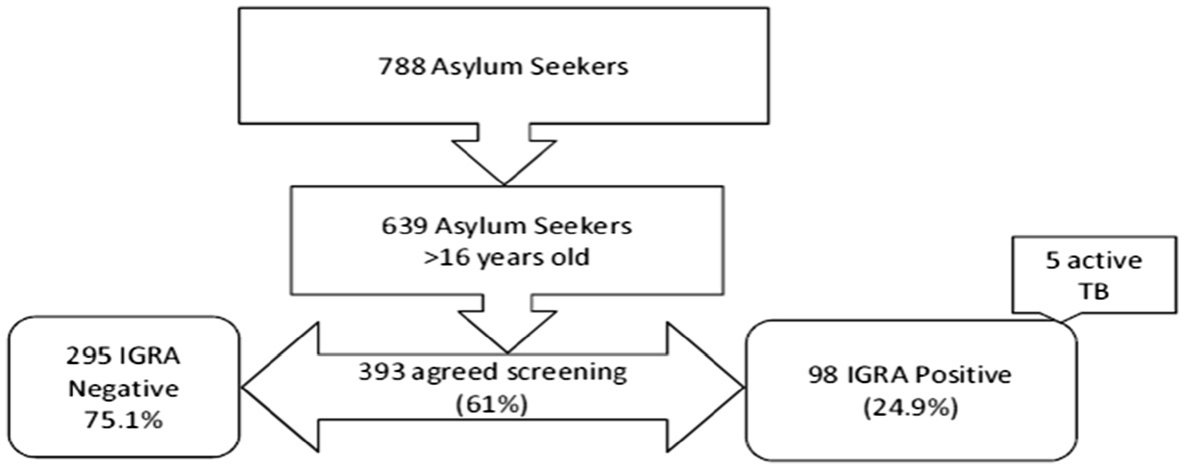
Study flowchart.

**Figure 2:**
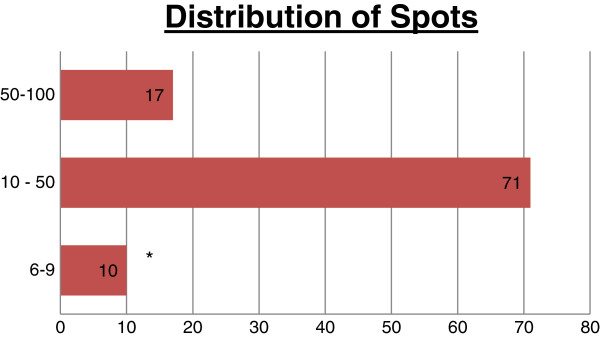
**Spot distribution among positive IGRA.** *During the study period we followed the actual Swiss Guidelines and the cases with 6–9 spots were counted as positive. The recent CDC recommendations differ and propose to consider tests results between 6 and 9 spots as borderline.

**Figure 3:**
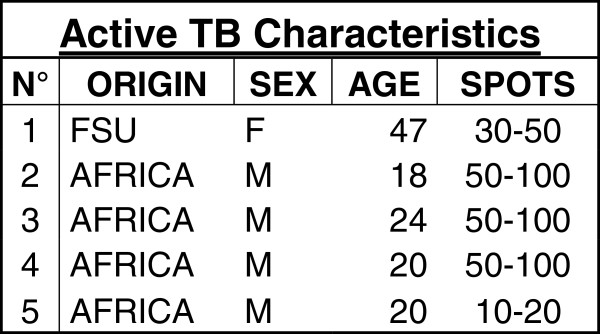
Characteristics of cases with active TB.

**Figure 4:**
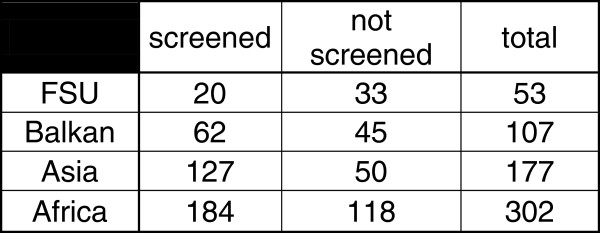
Distribution of the collective by region of origin.

Among 788 asylum seekers registered in two dedicated centers during the study period, 639 were adults over 16 years old. 393 of them agreed to be screened (61.50% of the eligible population). In this group, 295 (75.06%) asylum seekers had a negative T.SPOT.TB, and 98 (24.93%) had a positive T-SPOT.TB of which 5 (5.1%) had active tuberculosis (3 culture-proven) previously not detected at the border and 2 had already been treated for active tuberculosis prior to the entry in Switzerland (Figure 1). The spot distribution is provided on Figure 2 (Figure 2). The characteristic of the 5 cases with active tuberculosis are shown on Figure 3 (Figure 3). The remaining 38, 5% did not agree respond to the proposed screening or left the country before any investigation. Detailed data on the unscreened collective is scarce but their median age is of 29.26 years, 25% were women and their origin distribution is shown on Figure 4.

In the univariate analysis (Table 1) balkanic origin was set as reference since the tuberculosis incidence rate in balkanic countries is close to the western European countries. Associated with LTBI variables were: origin from FSU and Africa, ground/sea transit pattern, previous TB exposure and cough. The variables Age, Sex, Asian origin, being married, the existence of siblings and offspring as well as prior stay in congregate settings, addictions and immunosuppression were not associated with an increased risk of LTBI.

**Table 1:** Univariate analysis

**Factors**	**Positive n(%)**	**Negative n(%)**	**Odd ratio**	**(95% Conf. interval)**	**p<0.05**
**Age , mean(sd)**	29.09	27.63	1.02	0.99 , 1.04	0.179
**Age (by 10 years)**	29.09	27.63	1.18	0.93 , 1.49	0.179
**Sex**					
**Male**	214	72	1.05	0.62 , 1.76	0.858
**Origin**					
***balkanic origin (ref)***	54	8	ref.	ref.	ref.
***FSU***	12	8	4.5	1.41 , 14.4	0.011
***Asia***	110	17	1.04	0.42 , 2.57	0.927
***Africa***	119	65	3.69	1.65 , 8.22	0.001
**Ground transit**	109	60	2.49	1.54 , 4.02	0.000
**Married**	124	48	1.34	0.84 , 2.12	0.219
**Siblings**	243	73	0.53	0.30 , 0.92	0.025
**Offspring**	111	46	1.45	0.91 , 2.32	0.116
**Congregate settings**	96	34	1.1	0.68 , 1.78	0.695
**Addictions**	138	54	1.4	0.88 , 2.21	0.154
**Immunosuppresion**	19	7	1.3	0.46 , 2.78	0.79
**Prev. TB exposure**	12	9	2.38	0.97 , 5.84	0.057
**Cough**	17	12	2.3	1.05 , 5.01	0.036

The multivariate logistic regression (Table 2) identified origin from the Former Soviet Union 12.54 (2.02, 77.9), Asia 2.63 (0.49, 14.12) and Africa 26.11 (5.04, 135.43), ground transit 2.42 (1.34, 4.37), married status 2 (1.01, 3.82), and cough 8.08 (2.63, 24.87) as the major factors associated with LTBI. Prior TB exposure 1.94 (0.65, 5.72) and age by 10 years 1.37 (0.99, 1.88) were considered as minor factors related with LTBI because of their almost significant CI 95% interval.

**Table 2:** Multivariate logistic regression

**Risk factor**	**Odds ratio**	**(95% Conf. interval)**	**p<0.05**
**Age (by 10 years)**	1.37	0.99 , 1.88	0.054
**Origin**			
***balkanic (ref)***	ref.	ref.	ref.
***FSU***	12.54	2.02 , 77.9	0.007
***Asia***	2.63	0.49 , 14.12	0.26
***Africa***	26.11	5.04 , 135.43	0.000
**Ground transit**	2.42	1.34 , 4.37	0.003
**Married**	2	1.01 , 3.82	0.038
**Prev. TB exposition**	1.94	0.65 , 5.72	0.233
**Cough**	8.08	2.63 , 24.87	0.000

**Figure 5:**
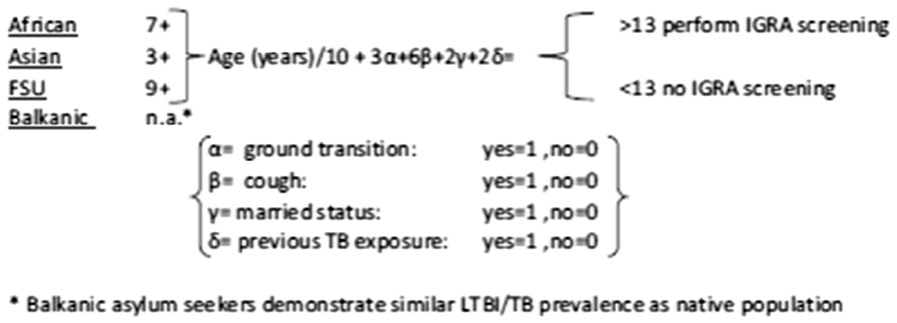
**Predictive score calculation.** Example: The score for an asylum seeker from former Soviet Union, aged 47 without ground transit, no cough, married with previous exposure to TB is equal to: 7+4.7+2+2=15.7. In such case, screening with IGRA is justified. *Balkanic asylum seekers TB prevalence as native population/TB prevalence as native population.

**Figure 6:**
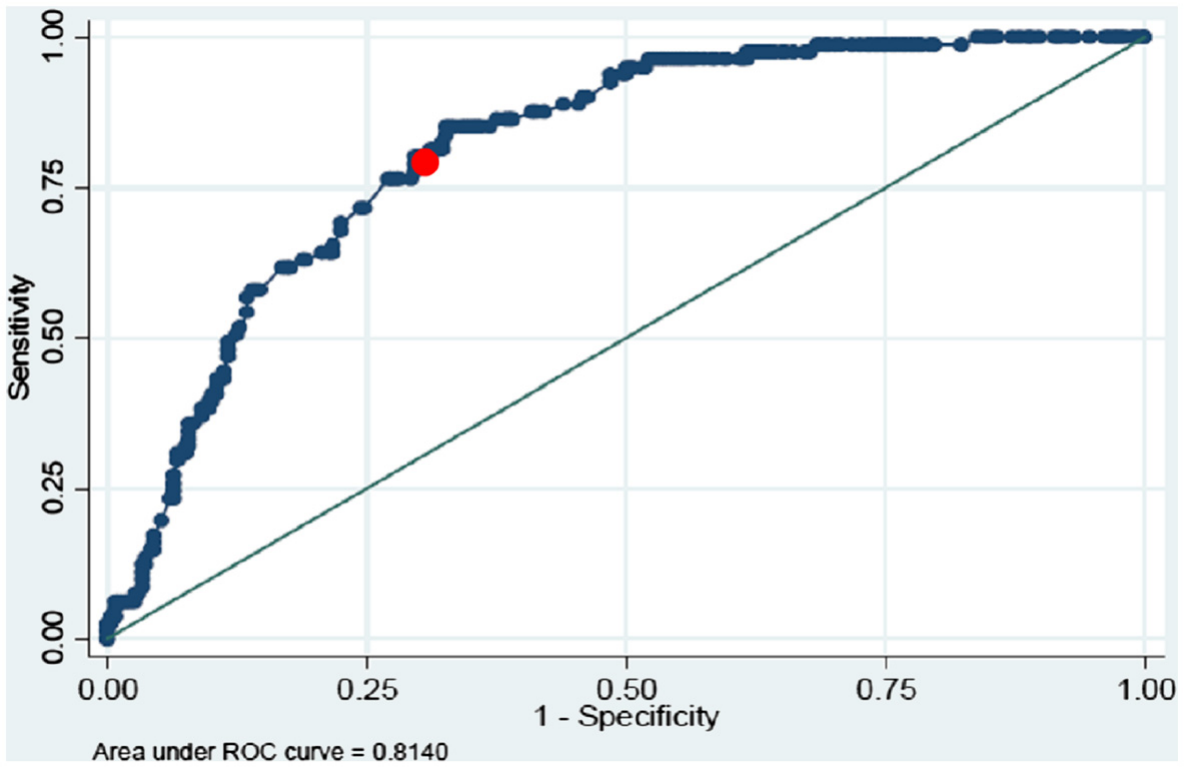
**The area under the ROC curve (AUC=81%) gives a measure of the discriminative power of the score between the infected and the control group.** The red dot corresponds to the sensitivity/specificity at a cut-off of 13.

The values of our predictive score (Figure 5.) ranged from 2 to 23 (median (11.5), IQR (6.3)). The probability of having a positive IGRA increases with score value. The score had a good discriminatory power (AUC=81%) with a sensitivity of 80%, a specificity of 70%, a PPV of 45% and a NPV of 92% when using the cutoff-score=13 (Figure 6).

## Discussion

The main objectives of this study were to assess the factors associated with LTBI among asylum seekers entering Vaud County. A robust score combining six factors (country of origin, travel conditions, age, marital status, cough, prior TB exposure) allowed the identification of AS with the highest risk of LTBI.

The prevalence of LTBI of 24.9% observed in this study among asylum seekers is close to the estimates reported in the literature. Winje and et al. reported a 29% [12] prevalence while Hardy AB and al, reported 38% [13]. Pareek [1] demonstrated that the proportion of asylum seekers in UK with a positive IGRA was between 3 and 28%, related to the incidence of tuberculosis in the home country and Mulder [9] reported a similar result with 20% positive QuantiFERON(®)-TB Gold In-Tube assay among AS.

A striking figure is the detection of 5 subjects with active tuberculosis that passed undetected through the border screening performed several weeks before. We assume that these subjects progressed from a recently acquired infection after border screening according to the natural history of TB. Considering the fact that the majority of cases of tuberculosis among asylum seekers are notified after entry, this is not surprising but underlines the fact that migrants with complaints or health problems should have rapid access to health care and tuberculosis diagnosis. A recent study by Ricks [14] highlighted the importance of LTBI screening and treatment in order to reduce the burden of TB among foreign born individuals in the US.

The multivariate logistic regression permitted to identify the major factors associated with LTBI. Married individuals from an African or an FSU country that crossed multiple borders to reach Switzerland border and who cough are mostly at risk of being infected. Two minor factors (age and positive history of TB exposure) were also highlighted. Using those six factors we elaborated a predictive model for screening asylum seekers for LTBI resulted in a score with an AUC=81%.

The risk of LTBI increases with age. Indeed the longer a person lives the greater are the risk of being in contact with an individual with active TB. The main feature of using age as LTBI predictor is the presumed time of infection. Due to their young age and to the travel conditions, frequently in very close contact with other persons during prolonged periods, we assume that these individuals have been infected recently.

In our study married individuals also had a higher risk of being infected with an odd ration of 2.0. This is a quite interesting finding since demographic data on disease shows an opposite relation [15]. Although no solid explanation can be given for this finding our collective showed a clear association with LTBI.

The prevalence of tuberculosis in the home country is correlated with a risk of having LTBI. The NICE guidelines suggest LTBI screening for all asylum seekers migrating from countries with a TB prevalence higher than 50/100.000 [11]. Applying this rule to Switzerland would mean screening the majority of asylum seekers and would imply high costs and logistical problems. Due to the limited population of this study the independent evaluation of each country of origin was not possible. Therefore we studied those countries mainly represented in the Swiss asylum seeker population. Bias due to the addition of populations like North Africans (low risk) and sub Saharan Africans (high risk) to the statistical analysis could not be avoided. Due to the absence of asylum seeker from Latin American origin in our collective, we could not assess the risk in this population.

The travel conditions to reach Switzerland were clearly related to the risk of LTBI infection. Individuals travelling directly to destination using airplane meet fewer migrants in their journey and therefore have a lower risk of TB infection. A long and hazardous journey through several borders using ground and/or sea transportation increases the risk of TB contacts and infection. Although the socioeconomic status of the asylum seeker might influence the travel pattern it is difficult to argue that ground/sea transit is less expensive than airplane but it is seems clear that access to airplane is limited to persons with higher socio-economic status and access to official (or fake) documents.

A previous exposure to TB is an obvious factor related with LTBI. A personal history of recent exposure to presumed or confirmed active TB person enhances the risk of LTBI and its reactivation potential mostly during the following two years.

That cough was identified as a risk factor for LTBI is surprising since by definition LTBI is an asymptomatic infection. This could be due to the fact that smokers (who are very prevalent in this population group) have a higher risk of LTBI and tuberculosis than non smokers [16]. Other plausible explanation for this finding could be the congregated way of living, especially during winter months in asylum seeker centers with high exposure to passive smoking as well as the lack of stratification during statistical analysis between chronic and acute coughing due to sample limitations. When present, chronic cough was extensively assessed to rule out disease while acute coughing was usually self-limited.

The limitations of our study are the local setting, the inherent characteristics of this mobile population, the cross sectional design and the voluntary pattern of enrolment. This study provided a realistic description of actual collective of asylum seekers arriving in Vaud county. As the asylum seeker population is randomly allocated in the different regions of Switzerland, we assume that this population group was representative of the demographic details of the whole asylum seeker population in Switzerland. Nevertheless in this study 61% of the recently arrived asylum seeker population was screened using IGRA qualifying this study as representative of the study population. In addition to this, since no selection was applied to the study population, the travel condition and characteristics of the asylum seeker entering Vaud County match those of asylum seekers entering in other western European countries. Some bias could result from the voluntary pattern of enrollment with an over-representation of sick migrants but this setting was essential for the ethical acceptance of the study protocol. As the proportion of migrants with positive IGRAs was similar as in comparable studies, we assume that this was not a bias. Moreover, among AS who entered in the centre, the actual number of eligible persons was lower since many of them left the territory or were rejected before the enrollment procedure could be started. We have decided to include all the asylum seekers that were present on the asylum seekers registry to the study collective to better describe the reality. Finally since this study is time and country specific the collective characteristics are subject to change over time following the shift in immigration pattern.

## Conclusions

This study highlights the factors associated with LTBI among asylum seekers entering Vaud County, Switzerland. The observed prevalence of LTBI (24.9%) matches with the prevalence from the literature. The prevalence of TB previously undetected at the border in asylum seekers with LTBI was also high (5.1%). The factors associated with LTBI identified in this population (age, origin from FSU, Asian and African countries, ground transportation; married status; prior TB exposure and cough) were combined to create a predictive score of LTBI for asylum seekers which could be used at border screening. The application of this score to an asylum seeker population could help discriminating those most at risk for LTBI permitting a limitation of the number of IGRA to be performed in a border screening setting.

## Abbreviations

LTBI: Latent tuberculosis infection; AS: Asylum seekers; TB: Tuberculosis; IGRA: Interferon Gamma Release Assay; AUC: Area under the curve; PPV: Positive predictive value; NPV: Negative predictive value; FSU: Former Soviet Union.

## Competing interests

No authors declared any competing interest in the performance of this study.

## Authors' contributions

AS acquired the study data and helped to the results interpretation, drafted the manuscript and revised it. JPZ conceived and designed the study, helped in the study coordination, interpretation of the results and revised the study manuscript. MF performed the statistical analysis and interpretation. OD participated in the study coordination. CD carried out the initial assessment and blood sampling; PB helped in the study coordination, contributed to the interpretation of results and revised the manuscript. All authors read and approved the final manuscript.

## Pre-publication history

The pre-publication history for this paper can be accessed here:

http://www.biomedcentral.com/1471-2334/12/285/prepub
